# Pharmacological Insights Into Ashwagandha (Withania somnifera): A Review of Its Immunomodulatory and Neuroprotective Properties

**DOI:** 10.7759/cureus.89856

**Published:** 2025-08-12

**Authors:** Satish P Dipankar, Mayuri M Dani, Roshni Anirudhan, Dinesh Tripathi, Chetna Mishra, Salam Himika Devi

**Affiliations:** 1 Physiology, All India Institute of Medical Sciences, Mangalagiri, Mangalagiri, IND; 2 Integrative/Complementary Medicine, Rasashastra and Bhaishajya Kalpana, Swami Vivekanand Ayurvedic Medical College and Research Center, Ahmednagar, IND; 3 Ayurveda Paediatrics, Government Medical College, Kerala University of Health Sciences, Thiruvananthapuram, IND; 4 Physiology, King George's Medical University, Lucknow, IND; 5 Life Sciences (Zoology) Genetics and Genetic Toxicology, Manipur University, Canchipur, IND

**Keywords:** adaptogen, ashwagandha, immunomodulation, neuroprotection, phytopharmacology, withania somnifera

## Abstract

*Withania somnifera*, commonly known as ashwagandha, is a cornerstone of traditional Ayurvedic medicine, widely revered for its adaptogenic, immunomodulatory, and neuroprotective potential. Science has recently provided more evidence that it supports the immune system and protects nerve functioning, which makes it a good choice for integrative and preventive medicine. This review summarizes the main active substances found in Ashwagandha, such as withanolides, alkaloids, and sitoindosides, as well as the way the body processes them. *W. somnifera*, also known as ashwagandha, is used in traditional Ayurvedic medicine because of its potent immunomodulatory, neuroprotective, and adaptogenic properties. By altering natural and acquired immunity, boosting natural killer cell activity, controlling T- and B-cell responses, and altering cytokine levels, ashwagandha is being researched for its potential to alter the immune system. Simultaneously, the plant's neuroprotective potential is investigated by taking into account its antioxidant properties, capacity to improve brain function, support for stress reduction, and potential to treat mental and neurological diseases. In addition, the article analyzes the latest medications, ongoing clinical tests, and safety details, noting the most common side effects, drug-herb interactions, and the point at which herbs become toxic. The discussion includes important gaps such as requiring standardized extracts, having more human data over time, and increasing molecular studies. The focus is now on combining genomic research, inventing new drug delivery systems, and making sure that ashwagandha-based interventions are used ethically in the market. Overall, this review consolidates multidimensional evidence on *W. somnifera*, underscoring its pharmacological value as a natural immunoneurotherapeutic agent and advocating for its broader inclusion in evidence-based healthcare models.

## Introduction and background

Background and traditional use

Ashwagandha, which is scientifically known as *Withania somnifera*, has long been an important medicine in Ayurveda and Unani for over 3,000 years. Ashwagandha has a long history of being seen as a Rasayana for its ability to rejuvenate, adapt, and support longevity [1]. According to the Charaka Samhita and Sushruta Samhita, ashwagandha is recommended for those suffering from weakness, tiredness, poor body weight, and neurological problems. Ashwagandha is classified in Unani as a "nervine tonic" and is used to help restore the body's vital humors [2]. Ashwagandha is usually made into churna (powder), avaleha (a paste or jam), kvatha (a decoction), or arishta (a fermented infusion). It is given as a mouth tablet, sometimes by itself or together with Shatavari, Brahmi, and Guduchi. The main ways to use these compounds are by adding them to milk or ghee or as ingredients in polyherbal compounds to improve the effects of different phytochemicals [3].

Scientific relevance in modern medicine

The movement towards using research-based medicine has made more people interested in trying ashwagandha. Because of its strong ethnopharmacological history and recent research, it is considered a valuable drug for treating neurodegenerative diseases, disorders of the immune system, stress, and metabolic problems [4]. Owing to progress in pharmacognosy and molecular biology, it is now known that ashwagandha contains withanolides, alkaloids, and sitoindosides, which support its traditional use. Interestingly, ashwagandha is being extensively researched in India and also in Western countries where phytotherapy and complementary medicine are becoming accepted in traditional healthcare. Many studies have focused on its ability to adapt, reduce inflammation, and influence hormones [5]. Ashwagandha is becoming more popular worldwide, as it is included in supplements, nutraceuticals, functional foods, and even products meant for increasing sports performance. Because of this surge, researchers are now able to explain the herb’s effects using science and data instead of just anecdotes.

Scope of the review

Our purpose in this review is to gather the current evidence that ashwagandha has effects on the immune system and brain protection. Although previous reviews have looked at the main pharmacological effects of cannabis, not many have specifically studied how it affects immunity and neural function, which are important for homeostasis and disease resistance [6]. This review uses molecular analysis, pharmacokinetics, clinical results, and study of formulation to provide a framework that connects traditional medicine with biomedical science. The review looks at issues such as differences in extracts, shortcomings in current clinical trials, and the requirement for new drug delivery systems. With immune-mediated and neurodegenerative illnesses increasing among aging and stressed populations, looking at ashwagandha’s role in treating these conditions is now both needed and important [7].

Objectives and research questions

This review aims to discover and describe the substances in *W. somnifera* that are responsible for its effects on the immune system and brain. Particular attention is given to examining how withanolides, sitoindosides, and alkaloids affect the immune system and the nervous system. The investigation aims to explore the possible mechanisms by which ashwagandha supports immunity, reduces oxidative stress, promotes brain cell growth, and manages brain inflammation. Combining the results of preclinical and clinical studies allows us to create a clear view of how ashwagandha might benefit the immune and nervous systems. To achieve these objectives, the review is based on a set of focused research questions. (1) What are the most important bioactive substances in *W. somnifera*, and how are they handled once they enter our body? (2) How does Ashwagandha help regulate the immune system and protect the brain? (3) How much evidence, both from experiments and from clinical studies, supports these health benefits? (4) What parts of current research need improvement, and which areas do we need to focus on next to help it be more useful in medicine?

**Figure 1:**
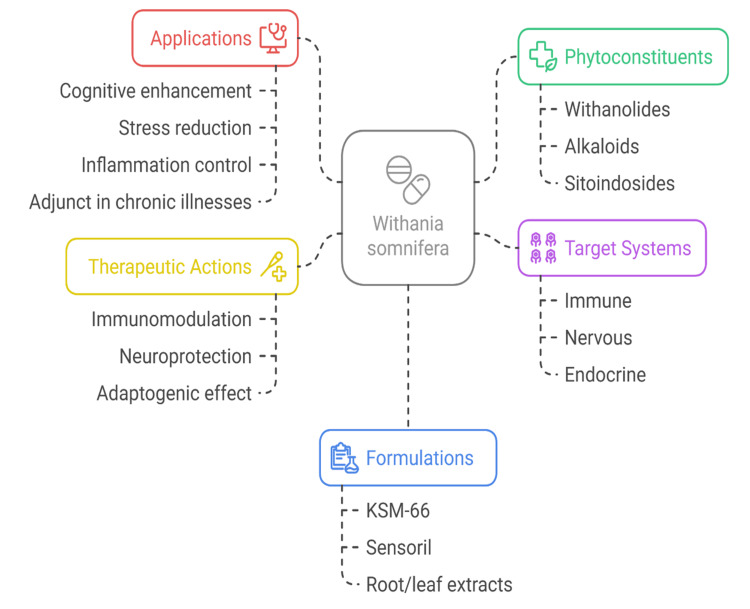
Pharmacological landscape of Withania somnifera KSM-66: Standardized Ashwagandha Root Extract Image credits: Satish P. Dipankar

Answering these questions helps explore ashwagandha as an effective herbal treatment in both prevention and therapy. Figure 1 displays a complete graph of *W. somnifera*’s pharmacological effects, identifying its active ingredients, their uses, and the systems they influence in modern and traditional medicine. 

## Review

Phytochemistry and pharmacokinetics of *W. somnifera*

Major Bioactive Constituents

Table 1 shows that *W. somnifera* leaves concentrate up to 1.60% withaferin A, several-fold higher than roots (≤ 0.08%), supporting leaf extracts when rapid immuno-neuro activity is desired [8,9]; roots instead provide a steadier profile that combines moderate withanolide A, most tropane alkaloids (0.13-0.31 %)and nearly all sitoindosides VII-X (< 0.20%), a constellation underpinning traditional anti-stress and immunostimulatory preparations [10]; aerial organs (leaf and stem) also supply about 4% total flavonoids that reinforce antioxidant defenses [11]; stem and seed tissues are chemically dilute yet offer trace withanolides useful for biomass-based production. Taken together, these organ-specific patterns justify blending selected leaf and root fractions to balance potent leaf withaferin A with the alkaloid-saponin stability of roots while underscoring the need for chemotype and harvest-time control to keep therapeutic batches within the indicated concentration ranges.

**Table 1:** Organ-wise distribution and typical concentration ranges of principal phytoconstituents in Withania somnifera (dry-weight basis) ^*^ Ranges compiled from multi-varietal field and chemotype studies; values shift with cultivar, plant age, and agro-climatic conditions. ^†^ Elite chemotypes reported up to 1.60% leaf withaferin A.

Constituents (key examples)	Predominant organ(s)	Typical concentration % w/w*	Representative references
Withaferin A (steroidal lactone)	Leaf ≫ Stem > Root > Seed	Leaf 0.13–1.60% †; Stem ≤ 0.19%; Root ≤ 0.08%; Seed ≈ 0.02%	[8,9]
Withanolide A (steroidal lactone)	Stem ≈ Root > Seed > Leaf	Leaf 0.0015–0.004%; Stem 0.003–0.025%; Root 0.003–0.015%; Seed 0.005–0.008%	[8,9]
Total tropane alkaloids (anaferine, anahygrine, cuscohygrine, isopelletierine)	Root ≫ Leaf/Stem	Root 0.13–0.31% (occasionally up to 4.3%); Leaf ≈ 0.09%	[10]
Sitoindosides VII–X (acyl-steryl/glyco-withanolide saponins)	Root > Leaf	Generally < 0.20%	[10]
Total flavonoids (quercetin-equivalent)	Leaf ≈ Stem > Root	Leaf 3.5–4.4%; Stem 4.2–4.4%; Root ≈ 3.9%	[11]

Apart from anolides, anaferine, anahygrine, cuscohygrine, and isopelletierine are major alkaloids that affect the pharmacology of this herb. Sitoindosides VII and VIII, together with withaferin A glycosides, have been shown to have anti-stress and immunostimulating effects [10]. Even though there are fewer of them, flavonoids in ashwagandha help get rid of reactive oxygen species (ROS) and backup systems that eliminate toxins [11]. Many of the phytoconstituents are found in the roots and leaves, but root extracts are often the main components used in medicine. Because of where, how, and when the ingredients are grown, the concentrations in batches can change and remain hard to control [12].

Pharmacokinetics and Metabolism

Knowing how ashwagandha’s important parts are handled by the body can help us use it more effectively. Even though withanolides are not easily soluble in water and get metabolized before they reach the bloodstream, they are only moderately absorbed orally and have low bioavailability [13]. After absorption, withanolides move to a number of organs, but they are primarily found in the liver and brain. These substances are known to be metabolized by the liver via cytochrome P450 enzymes, after which other routes add glucuronic acid or sulfate [14]. Kidneys and the liver handle most excretion, but the time it takes varies a lot, depending on both the medicine and what is taken along with it. One important issue in ashwagandha pharmacology is that it is not easily taken up by the body. Researchers are trying to address these problems by combining piperine, using liposomes, or developing nanoemulsion formulations [15].

Standardization of Extracts

A lot of ashwagandha extracts sold today vary in their phytochemicals and how powerful they are. KSM-66 and Sensoril are two standardized preparations that are widely studied and confirmed by clinical research. KSM-66 is created by water extraction, which helps preserve the natural mixture of its compounds. Most often, adaptogenic herbs are suggested to help with stress, endurance, and brain health due to their withanolide content [16]. The main source of Sensoril is from the leaves and roots, and its high withanolides (often 10% or more) lead to its use in supporting heart and metabolic health. Apart from using butanolic extracts, experiments also use hydroethanolic and methanolic extracts, and these differ both in the amount of withanolides and their performance [17]. How cannabis is processed has a major effect on its chemical content. When extracting bioactives, the polarity of the solvent, how hot it is, and how long the extraction takes all matter. That is why quality control needs to be very strict, and all batches should be standardized.

Analytical Techniques

Effective analysis methods are required to standardize and properly identify extracts from *W. somnifera*. Withanolides and alkaloids can be measured in both unprocessed and processed plants by using high-performance liquid chromatography (HPLC) [18,19]. Lab technicians often rely on liquid chromatography-mass spectrometry (LC-MS) and nuclear magnetic resonance (NMR) spectroscopy as a pair for identifying and detecting metabolites [20]. Using these methods, it becomes possible to assess both the presence and amount of individual phytochemicals. To check the purity and look for contaminants, thin-layer chromatography (TLC) and gas chromatography (GC) are used as additional support. Together, these methods help make ashwagandha products more reliable in both scientific and clinical settings [21].

Immunomodulatory properties of ashwagandha

Effects on Innate Immunity

Ashwagandha has a powerful effect on the body’s natural immune system. Research in animals has found that ashwagandha extracts increase the ability of macrophages to engulf pathogens, boost the proliferation of natural killer (NK) cells, and turn on dendritic cells [22].

These results are achieved by changing how pattern recognition receptors work and by strengthening the body’s ability to fight pathogens. Ashwagandha stimulates neutrophils to release oxidative bursts and encourages lysosomal enzymes, which help clear pathogens more quickly. The herb is also involved in hematopoiesis, which supports the innate immune system, when useful for a long time [23].

Modulation of Adaptive Immune Response

Ashwagandha has been proven to help raise T-cell numbers and to support a good ratio of T-helper cell types in the adaptive immune system. It helps stop the immune system from becoming either too active or too weak. In mice, taking ashwagandha raised the numbers of CD4+ and CD8+ T-cells, indicating that it might help with both helper and cytotoxic immune responses [24]. Ashwagandha is thought to encourage B-cells to activate and produce immunoglobulins, which may raise the strength of humoral immunity. In autoimmune models, the herb helps by lowering Th17 activity and raising T-regulatory activity [25].

Cytokine Regulation and Inflammatory Pathways

A key part of ashwagandha’s immunomodulatory effect is its ability to control the levels of two important cytokines. It reduces the amount of tumor necrosis factor-alpha (TNF-α), interleukin 1-beta (IL-1β), and interleukin 6 (IL-6) in the body, and at the same time, it increases interleukin 10 (IL-10) [10,11]. Nuclear factor kappa-B (NF-κB) signaling and Janus kinase/signal transducer and activator of transcription (JAK/STAT) signaling are blocked by this cytokine-balancing effect. Ashwagandha helps lower inflammation in the body by decreasing the levels of cyclooxygenase-2 (COX-2) and inducible nitric oxide (NO) synthase (iNOS). Such regulation is helpful during both acute immune reactions and persistent inflammatory and autoimmune diseases, acting as a safer substitute for synthetic immunosuppressants in some cases [12].

Experimental and clinical evidence

In vitro and in vivo research is finding that ashwagandha can regulate the immune system. When human lymphocytes and macrophages are treated with ashwagandha extracts, both their survival and the amount of cytokines produced go up [13]. The herb has been shown to improve how animals resist infections from bacteria, viruses, and fungi. In the case of rheumatoid arthritis, multiple sclerosis, and type 1 diabetes, ashwagandha is connected to better symptoms and fewer autoantibodies [14].

**Figure 2:**
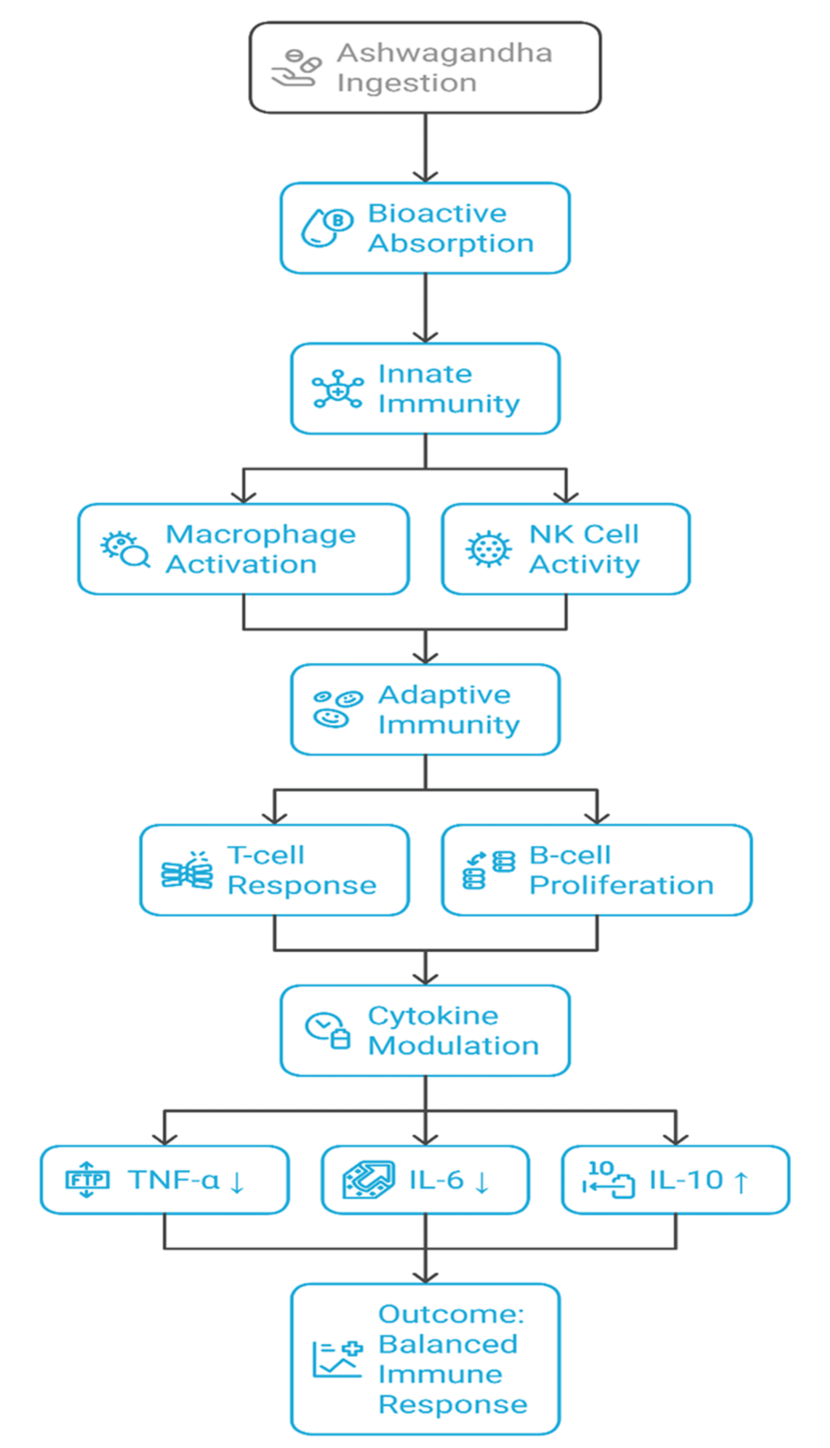
Immunomodulatory mechanisms of ashwagandha NK Cell: Natural Killer Cell; TNF-α: Tumor Necrosis Factor Alpha; IL-6: Interleukin 6; IL-10: Interleukin 10 Image credits: Satish P. Dipankar

The same effects are observed in models of infection, where ashwagandha improves vaccine results and speeds up pathogen removal. Improvements in white blood cell counts, NK cell activity, and general markers of the immune system have been observed in clinical trials with immunocompromised individuals undergoing chemotherapy or having chronic infections [15]. Even though the findings are encouraging, many studies are still quite small, so additional randomized trials are necessary to confirm them and decide on the best dosage. The diagram in Figure 2 outlines the way ashwagandha acts on the immune system by boosting immunity, influencing immune cells, and controlling cytokines according to the results of current research.

Neuroprotective mechanisms of ashwagandha

**Figure 3:**
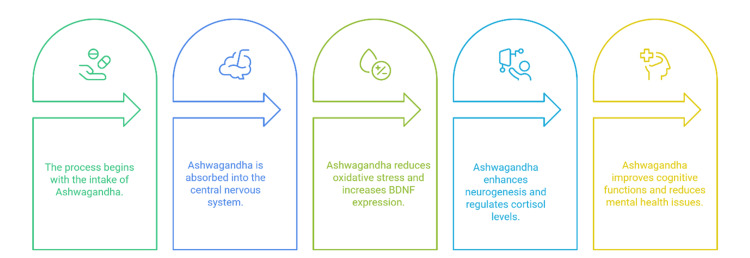
Neuroprotective pathways of ashwagandha BDNF: Brain-Derived Neurotrophic Factor Image credits: Satish P. Dipankar

Figure 3 shows that ashwagandha works in several ways, including antioxidant effects, supporting the growth of new brain cells and control of stress by modulating the hypothalamic pituitary adrenal (HPA) axis.

Antioxidant and Anti-inflammatory Actions in the Brain

*W. somnifera* (ashwagandha) is known to protect the nervous system largely because of its powerful antioxidant and anti-inflammatory properties. The herb helps reduce the formation of ROS and NO, which are thought to play a role in Alzheimer’s and Parkinson’s diseases [20]. It also protects the integrity of both cellular membranes and mitochondria by reducing lipid peroxidation in the brain [21]. At the molecular level, ashwagandha acts to reduce NF-κB activation, which is a main transcription factor involved in brain inflammation. In addition, lowering COX-2 activity helps reduce prostaglandin production, which lowers inflammation throughout the central nervous system (CNS) [22]. These effects combined reduce damage to neurons, help cells become stronger, and maintain healthy brain function [23].

Cognitive Function and Memory Enhancement

Ashwagandha is often studied because it may help with memory and mental function, so it is considered a good choice for age-related mental decline and attention-deficit problems. According to preclinical data, withanolides boost brain plasticity, allow for better long-term potentiation, and improve how well animals perform in tasks that require spatial memory [24]. Some of these modifications are due to higher levels of brain-derived neurotrophic factor (BDNF), a neurotrophin that helps neurons grow, live, and develop [25]. In addition, ashwagandha helps create new nerve cells in the hippocampus, which is important for learning and memory, especially as people get older. Cholinergic signal transmission is improved when acetylcholinesterase function is changed, which is necessary for healthy attention and memory [26]. Researchers have found that healthy adults and patients with mild cognitive impairment show better working memory, executive function, and processing speed after taking these drugs in clinical trials [27].

Adaptogenic and Anti-Stress Effects

Its main power to protect the brain comes from its adaptogenic properties. Ashwagandha helps stabilize the body’s functions under ongoing stress by affecting the hypothalamic-pituitary-adrenal axis. The plant’s action in reducing serum cortisol limits the damage that chronic stress can have on your brain [28]. Ashwagandha also shows anxiolytic and antidepressant effects in laboratory animals that are similar to those of lorazepam and imipramine. This may happen because it impacts gamma-aminobutyric acid (GABA) signaling, alters monoamine pathways, and blocks the damaging effects of glutamate on the brain under stress [29]. Taking ashwagandha is associated with lower anxiety, fewer sleep disturbances, and less mood disturbance, suggesting it might benefit people with stress-related neurological diseases [30].

Therapeutic Potential in Neurological Disorders

Researchers are finding more evidence that ashwagandha helps treat and prevent various neurological disorders. Ashwagandha has been found to help reduce amyloid plaques, restore connections between brain cells, and enhance how people with Alzheimer’s behave [31]. Parkinson’s disease shows that curcumin maintains dopamine-producing cells and reduces the effects of oxidative stress [32]. Ashwagandha seems to help with seizures by controlling how much neurons are stimulated and the seizure threshold, owing to its effect on GABA. Additionally, research has found that the herb affects the HPA axis and helps people resist learned helplessness in models of major depressive disorder [33]. Current findings indicate that cannabidiol (CBD) may support neuro-restoration after a traumatic brain injury by helping with axon regrowth and remyelination [34].

Therapeutic applications and clinical utility

Commercial Formulations and Dosage Regimens

Many commercial varieties of ashwagandha are sold in capsule, tablet, powdered root (churna), and tincture form. Among all, the most common form of avoiding usage in research and therapy is root extract standardized to have 1.5-10% withanolides [35]. Most adults are prescribed 300-600 mg each day of a standardized extract, but sometimes higher amounts are given under a doctor’s supervision. How much CBD you take should be determined by your intended results - low doses are enough for wellness and relaxation, but higher doses help treat disorders such as multiple sclerosis or Alzheimer’s [36]. Researchers prefer to study KSM-66 and Sensoril due to their high quality and dependable results. KSM-66 enhances energy and mental function, and Sensoril has a stronger ability to lower stress, all due to its high withanolide content [37].

Ashwagandha in Integrative and Preventive Medicine

Ashwagandha is increasingly popular as a natural part of preventive and complementary healthcare. Given the many positive effects it has, it often accompanies yoga, meditation, and adaptogenic drugs such as Rhodiola and Bacopa to make a person’s mind calmer and enhance brain health and memory [38]. Taking ashwagandha is commonly done to slow down age-related problems with the immune system and brain health in those at risk of neurodegeneration. Because it can help manage stress, it is well-suited for psychosomatic illnesses, burnout, and conditions related to poor lifestyle [39]. Since it works well in most cultures and has few side effects, ashwagandha links age-old treatments with scientific evidence. Taking adaptogens in a wellness program is often done to get better sleep, less fatigue, and overall increased energy [40].

Clinical Trials and Translational Outcomes

Many randomized controlled trials (RCTs) have measured how ashwagandha can boost thinking, reduce stress levels, and improve immunity. Adults experiencing chronic stress who took KSM-66 (300 mg) each day for two months had lower cortisol levels and better well-being [25]. A second clinical trial found that people who took ashwagandha had better memory and attention test results, and their immediate recall and how they processed information improved significantly [27]. Increased NK cell activity and immunoglobulin A (IgA) secretion observed after vaccine trials suggest that the immune system is better protected at the sites where mucous membranes exist [24]. Most of the studies show moderate-to-large effects, but many have short sample sizes, short lengths of study, and no long-term tracking. However, the collected research supports much of what ashwagandha is known for and suggests it can be useful in modern therapy [30].

Target Populations and Indications

Ashwagandha appears to assist people with poorly functioning immune and nervous systems. It supports cognitive flexibility, memory, and mood, which better the quality of life for patients. Because of its immune-boosting powers, it also helps people who have cancer or a chronic viral infection, as their immune systems are weak [33]. For people with mental illnesses, ashwagandha is frequently used with antidepressants and anxiolytic drugs, mainly to help with anxiety, generalized anxiety disorder, and post-traumatic stress disorder when other treatments do not work well. The use of statins to stop the deterioration of memory and mental health in people who are at increased risk is currently being studied [34]. For these same reasons, people are studying ashwagandha to see how it can support children, athletes, and those who have unpredictable schedules by keeping their body rhythms balanced, building stamina, and preventing fatigue. As a result, it is a key element in both preventive and individualized health strategies [35]. We have provided a summary of how each drug affects the body, what proteins it works on, and so on in Table 2.

**Table 2:** Summary of pharmacological actions, target systems, mechanisms, clinical relevance, and supporting references of Withania somnifera NK cells: Natural Killer Cells; ROS: Reactive Oxygen Species; TNF-α: Tumor Necrosis Factor Alpha; IL-6: Interleukin 6; IL-10: Interleukin 10; BDNF: Brain-Derived Neurotrophic Factor; NF-κB: Nuclear Factor Kappa-Light-Chain-Enhancer of Activated B Cells; HPA Axis: Hypothalamic–Pituitary–Adrenal Axis; ACh: Acetylcholine; T3: Triiodothyronine; T4: Thyroxine; GABAergic: Refers to Gamma-Aminobutyric Acid-Mediated Neurotransmission; Nrf2: Nuclear Factor Erythroid 2-Related Factor 2; HO-1: Heme Oxygenase 1; IgA: Immunoglobulin A; MCI: Mild Cognitive Impairment

S. No.	Pharmacological Action	Target System	Mechanism of Action	Form/Extract Used	Clinical Implication	Reference(s)
1	Immunomodulation	Immune	↑ NK cells, ↑ macrophages	Root extract	Enhances immune defense	[22,24]
2	Antioxidant	CNS	↓ ROS, ↓ lipid peroxidation	Hydroethanolic extract	Protects neural integrity	[20,21]
3	Anti-inflammatory	Immune, CNS	↓ TNF-α, ↓ IL-6, ↑ IL-10	KSM-66	Reduces inflammation	[10,23]
4	Neuroprotection	CNS	↑ BDNF inhibits NF-κB	Sensoril	Slows neurodegeneration	[25,28]
5	Adaptogenic	Endocrine/CNS	↓ Cortisol, HPA axis balance	Root powder	Reduces stress response	[28,30]
6	Cognitive enhancement	Brain	↑ Neurogenesis, ↑ ACh activity	Ethanolic extract	Improves memory, focus	[24,27]
7	Antidepressant	CNS	Modulates monoamines	Full-spectrum extract	Mood stabilization	[29,33]
8	Thyroid modulation	Endocrine	↑ T3/T4 synthesis	Root extract	Caution in hyperthyroidism	[34]
9	Anti-seizure	CNS	↑ GABAergic tone	Methanolic extract	Reduces seizure threshold	[33]
10	Anticancer (adjunctive)	Systemic	Immunopotentiation, cytoprotection	Withaferin A	Supports chemotherapy	[16,20]
11	Memory aid	CNS	↑ hippocampal function	Sominone™	Enhances cognition in MCI	[25]
12	Hepatoprotective	Liver/CNS	Activates Nrf2/HO-1	Ethanol root extract	Prevents neurotoxic liver damage	[21]
13	Formulation support	Systemic	Improved bioavailability	Nano-emulsions	Enhances CNS delivery	[15]
14	Immunoglobulin stimulation	Immune	↑ IgA, ↑ B-cell activation	Sensoril	Prevents mucosal infections	[24,25]
15	Prophylactic support	Geriatrics	Broad-spectrum immuno-cognitive boost	KSM-66 capsules	Prevents aging-related decline	[36,37]

Safety profile and toxicological considerations

Acute and Chronic Toxicity Studies

Many preclinical studies have proven that *W. somnifera *has a high safety level and is hard to overdose. When used at the recommended therapeutic doses, the LD₅₀ of biotin in rodents is wide, ranging between 1,260 and 2,000 mg/kg [30]. No deaths or changes in behavior were observed in mice given high doses in acute studies, and no significant changes were seen in organs or blood after 90 days of repeated dosing [31]. Toxicology tests found that the medicine has a minimal impact on the liver, kidneys, and heart. However, slightly higher liver enzymes at strong doses suggest that it is important to watch doses in patients with liver problems. These tests also revealed that standardized ashwagandha extracts do not cause cancer.

Side Effects and Contraindications

*W. somnifera* is tolerated by the majority of people. A recent prospective, randomized, placebo-controlled trial that specifically assessed its safety found no serious adverse events; the only complaints were mild, transient gastrointestinal discomfort, including nausea and flatulence [20]. This margin of safety is provided by pre-clinical data: oral LD_50_ values in rodents are greater than 1 g kg-1, and only minor, reversible increases in liver enzymes were observed following 90-day, high-dose regimens [30,31]. Adverse effects are clinically insignificant but must be separated out as possible complications of these side effects; that is, thyroid-hormone stimulation may worsen hyperthyroidism [33,34], and the immunostimulatory action of the herb may provoke autoimmune diseases such as lupus, rheumatoid arthritis, or multiple sclerosis [35]. The isolated reports of uterine-contractile activity mean that there is a discouragement of use during pregnancy and lactation until more robust human data are available [36]. In general, modern evidence suggests a broad therapeutic index, although these absolute contraindications and the necessity of dose-regulating in hepatic impairment are to be taken into account in clinical practice.

Herb-Drug Interactions

How ashwagandha interacts with traditional medicines adds another problem to consider. Because it is an immunomodulator, it could make immunosuppressive agents such as corticosteroids and calcineurin inhibitors less effective in patients who have had transplants [37]. If taken together, alcohol can increase the sedative effects of antidepressants, benzodiazepines, and antiepileptics, as well as affect the way the body processes these drugs [38]. The herb can also influence hepatic enzyme systems, mainly cytochrome P450 isoenzymes such as CYP3A4 and CYP2D6, which help break down many different drugs. As a result of this modulation, warfarin, digoxin, and SSRI drugs in the plasma may need to be checked regularly [39]. Thus, it is necessary to monitor the use of ashwagandha when it is taken along with multiple other medicines, especially by older or chronically ill people [40].

Regulatory and Quality Control Aspects

Around the world, the way ashwagandha is regulated is changing. Ashwagandha is considered a traditional medicinal plant by the WHO, but it advises that more standardization is needed to guarantee its safety and effectiveness [41]. Ashwagandha is listed as a dietary supplement in the United States owing to the Dietary Supplement Health and Education Act (DSHEA), and it is the manufacturers’ responsibility to ensure they follow good manufacturing practices (GMP). A big challenge is standardization, because differences in withanolide content, extraction methods, and the materials used can result in uneven effects on the body. The FDA and EMA point out that analytical techniques must be validated to ensure a product is safe and as described [42]. Ensuring quality standards is important, so methods such as HPLC or LC-MS must be validated and used to reduce batch variability for consumer safety [43].

Research gaps and limitations

Variability in Extracts and Standardization Issues

Non-standardization is a major restriction not only to *W. somnifera* but also to the majority of nutraceuticals and herbal supplements. The variability is particularly critical in the case of Withania because of the varying pharmacological profiles of parts of the plants. Such examples include leaves, which contain significantly higher levels of withaferin A - a known cytotoxic and anti-tumor agent than roots, whose withaferin A is lower but has more equitable adaptogenic and immunomodulatory effects [44]. Although withaferin A can enhance anti-cancer potential, its cytotoxic property makes it unsafe when misused or taken in excess. This highlights the significance of root-only extracts being used in the majority of therapeutic preparations, and this has been the principle of traditional Ayurvedic medicine. Most commercial preparations are, however, not well defined in the plant part utilized and can contain whole-plant or leaf-based extracts that add the risk of unintended adverse effects. Furthermore, the variability in extraction solvents (aqueous vs. alcoholic), the concentrations of withanolides, and the batch-to-batch differences depending on the time of harvesting, harvesting site, and the processing procedures also make it difficult to interpret the clinical data [44]. Even though some quality assurance is provided by the use of standardized extracts such as KSM-66 (roots, aqueous) and Sensoril (roots + leaves, hydroalcoholic), such are not consistently used in trials, and meta-analysis and harmonization of doses are challenging [45]. Therefore, the priority of future studies ought to be based on root-only standardized formulations, extraction methods should be disclosed, and standardized analytical procedures should be adopted to be both safe and reproducible.

Methodological Limitations in Studies

Despite the limitations on the results of early trials of *W. somnifera*, including small sample size, brief duration, or absence of a placebo, in recent years, randomized, placebo-controlled clinical trials have increased, some of which report both efficacy and safety data in well-defined populations [46]. However, heterogeneity still exists: some studies do not stratify the participants by such important variables as age, sex, and comorbidity, which precludes the generalizability of the results. In addition, several are extremely dependent on subjective results, such as perceived stress or quality-of-life ratings, without utilizing validated biochemical or neuroendocrine measures. The next point of concern is the lack of consistency or completeness in reporting adverse events, a more accurate term than adverse effects, that could result in an underestimation of possible risks of using ashwagandha in long-term use or high doses [30]. Future research should be conducted following the CONsolidated Standards of Reporting Trials (CONSORT) guidelines and should include a powerful statistical design and multicentric designs in order to enhance clinical relevance and safety assurance. Positively, national organizations such as Indian Council of Medical Research-National Institute of Traditional Medicine (ICMR-NITM) have started systematic RCTs to produce evidence-based data of high quality on standardized extracts of plants, such as *W. somnifera*, thereby helping in scientifically verifying the traditional medicines [46].

Insufficient Long-Term and Human Data

Although ashwagandha has been studied in animals and short-term trials, not many long-term studies have tested how safe and effective it is in humans. It is unclear how chronic use of COVID-19 vaccines affects the elderly and people with weakened immune systems. Moreover, there is not enough pharmacovigilance data available. The combination of studies, continuous monitoring, and pharmacodynamic processes is necessary to establish safety over the long term. Follow-up periods of 6-12 months or more in cohort studies are needed to address this gap.

Challenges in Translational Research

Although *W. somnifera* is getting more attention, there are several barriers to its smooth conversion into clinical practice. The most important is the great variability of plant source and composition, which is affected by geographic, seasonal, and processing factors [44]. Such variation renders extracts hard to standardize either to obtain regulatory approval or clinical reproducibility. Moreover, most of the active constituents of the ashwagandha plant are naturally present and therefore cannot be patented, which provides no major commercial incentive to conduct large-scale, industry-funded studies. A knowledge gap in the molecular mechanism of the pharmacological effects of single constituents, as withaferin A, withanolide A, and sitoindosides, is one of the most critical bottlenecks. Although these compounds show promising neuroprotective, immunomodulatory, and adaptogenic properties, the signaling mechanisms and specific targets are not fully characterized, which restricts the development of mechanism-based treatments [14,15].

Moreover, the pleiotropic nature of the herb, which engages several physiological systems and receptor networks, makes the clinical positioning and regulatory classification of the herb difficult. In addition to scientific issues, other translational challenges are the lack of funds, unaddressed intellectual property concerns, and ethical considerations of bioprospecting and traditional knowledge owner rights. To solve these problems, it will be necessary to collaborate interdisciplinary with ethnopharmacologists, molecular scientists, regulatory agencies, and public organizations - connecting folk wisdom and contemporary biomedical paradigms.

Future directions and recommendations

Strengthening Clinical Evidence through Long-Term Randomized Trials

Although several randomized, placebo-controlled clinical trials on *W. somnifera* have already been performed and registered at such platforms as CTRI (India) and ClinicalTrials.gov [46], additional large-scale, multi-center trials are needed. These should be methodologically rigorous, that is, randomized, double-blind, and placebo-controlled trials, but also have good stratification by age, sex, ethnicity, and comorbid conditions to enhance translational value. The future research is expected to have longer follow-up periods (612 months) and include subjective and objective outcome variables. The therapeutic potential of ashwagandha should be measured using immune biomarkers, inflammatory cytokine profile, neuroendocrine markers, cognitive performance parameters, in addition to psychological indices such as the perceived stress scale (PSS) and Beck depression inventory (BDI).

Advancing Molecular, Genomic, and Systems Biology Research

The particular molecular mode of action of the various components of *W. somnifera* is very poorly understood. It is recommended that future studies increase the application of high-throughput omics approaches, not only genomics and transcriptomics but also proteomics, metabolomics, lipidomics, and epigenomics. Such strategies can give us a clue on how withanolides and other bioactive compounds regulate the expression of genes that are involved in immunity, stress regulation, inflammation, neuroplasticity, and metabolic regulation. To explain long-term regulatory effects, epigenetic profiling might be used, and systems biology/integrative pathway analysis can be used to explain the multi-target nature of this adaptogenic herb. This kind of mechanistic data will be useful in defining pharmacodynamic markers and in developing precision phytotherapy strategies.

Novel Formulation and Drug Delivery Approaches

Future formulation approaches to address the low bioavailability and target site delivery, particularly in the case of CNS actions, need to be directed at sophisticated drug delivery systems. They may be nanoparticles, liposomes, phytosomes, solid lipid carriers, mucoadhesive gels, and transdermal systems that have the potential to enhance the pharmacokinetic profile and therapeutic index of ashwagandha extracts. Even in the preclinical studies, the targeted delivery systems are already proving fruitful, especially in terms of neuroprotective efficacy and the reduction of adverse effects associated with systemic exposure. The technologies also provide an opportunity for dosage reduction and increased standardization among the product lines.

Standardization, Regulatory Oversight, and Ethical Policy Frameworks

Non-standardization is a usual issue in the areas of herbals and nutraceuticals, and *W. somnifera* is not an exception. The different composition of extracts based on different parts of the plant is one of the critical issues. As an example, leaves have greater concentrations of withaferin A, a substance that has strong anti-tumor but also cytotoxic effects, and roots have low levels and are safer to use in the long term [44]. Nevertheless, whole-plant extracts are present in many commercial preparations, but their origin is not clearly mentioned and may cause unintentional side effects. Future products, then, should meet root-only extract requirements and should be clearly labeled and well-certified in quality.

Simultaneously, the policy framework that regulates the usage of *W. somnifera* should be able to support safety, sustainability, and ethical bioprospecting. Enforcement of compulsory labeling, reporting of adverse events, and evidence-based health claims should be the mandate of the regulatory agencies. Moreover, it is essential to make sure that communities, which provide the traditional knowledge, are given appropriate recognition and benefit-sharing, as it is outlined by the Nagoya Protocol. The commercial value chain of the plant should be integrated with sustainable agricultural methods, fair trade certification, and biocultural conservation strategies so that its responsible global utilization could be ensured.

## Conclusions

The review showed that *W. somnifera* (ashwagandha) has immunomodulatory and neuroprotective effects because of its phytochemicals, including withanolides, alkaloids, and sitoindosides. By strengthening the immune system’s natural and learned responses, boosting the functions of macrophages and NK cells, controlling cytokine levels, and preserving immune tolerance, ashwagandha becomes a powerful natural immunotherapeutic. Furthermore, its ability to stop oxidation, fight inflammation, and adjust to stress helps protect the brain, improve thinking skills, encourage new brain cells in the hippocampus, and control the stress response via the HPA axis. Such actions make ashwagandha an attractive herb for use in integrative health plans that treat conditions involving inflammation and stress.

Even though an increasing amount of evidence is backing the therapeutic potential of *W. somnifera*, before it can be generally accepted as a mainstream therapeutic agent, large-scale, well-designed clinical trials are necessary. The state of the current research is hindered by methodological differences, the inconsistency in the formulation of extracts, and the lack of knowledge of its molecular mechanisms. The complete potential of ashwagandha is yet to be unlocked, and it would be achieved through the combined efforts of high-tech omics technologies, optimal drug delivery systems, and ethically responsible commercialization approaches. However, the current body of evidence is very convincing of its duality, both as a traditional remedy with millennia of history, as well as a scientifically proven agent with potential immunomodulatory and neuroprotective properties in contemporary medicine.
